# Factors associated with weight gain during COVID-19 pandemic: A global study

**DOI:** 10.1371/journal.pone.0284283

**Published:** 2023-04-20

**Authors:** Junjie Huang, Sze Chai Chan, Samantha Ko, Harry H. X. Wang, Jacky Yuan, Wanghong Xu, Zhi-Jie Zheng, Hao Xue, Lin Zhang, Johnny Y. Jiang, Jason L. W. Huang, Ping Chen, Erlinda Palaganas, Pramon Viwattanakulvanid, Ratana Somrongthong, Andrés Caicedo, María de Jesús Medina-Arellano, Jill Murphy, Maria B. A. Paredes, Mellissa Withers, Martin C. S. Wong, On behalf of the Association of Pacific Rim Universities (APRU) Global Health Program

**Affiliations:** 1 JC School of Public Health and Primary Care, Faculty of Medicine, The Chinese University of Hong Kong, Sha Tin, Hong Kong; 2 Centre for Health Education and Health Promotion, Faculty of Medicine, The Chinese University of Hong Kong, Sha Tin, Hong Kong; 3 School of Public Health, Sun Yat-Sen University, Guangzhou, China; 4 The Seventh Affiliated Hospital, Sun Yat-Sen University, Guangzhou, China; 5 Department of Epidemiology, School of Public Health, Fudan University, Shanghai, China; 6 Department of Global Health, School of Public Health, Peking University, Beijing, China; 7 Center for Experimental Economics in Education, Shaanxi Normal University, Xi’an, China; 8 School of Population Medicine and Public Health, Chinese Academy of Medical Sciences & Peking Union Medical College, Beijing, China; 9 North Ruijin Hospital, Shanghai Jiaotong University, Shanghai, China; 10 Institute of Management, University of the Philippines Baguio, Baguio, Philippines; 11 College of Public Health Sciences, Chulalongkorn University, Bangkok, Thailand; 12 Instituto de Investigaciones en Biomedicina iBioMed, Universidad San Francisco de Quito, Quito, Ecuador; 13 Institute of Legal Research, National Autonomous University of Mexico (UNAM), Mexico City, Mexico; 14 Department of Psychiatry, Faculty of Medicine, The University of British Columbia, Vancouver, Canada; 15 i3S-Instituto de Investigação e Inovação em Saúde, Universidade do Porto, Porto, Portugal; 16 Department of Population and Public Health Sciences, Keck School of Medicine of USC, University of Southern California, Los Angeles, CA, United States of America; University of Catania: Universita degli Studi di Catania, ITALY

## Abstract

**Background:**

The coronavirus disease 2019 (COVID-19) pandemic has resulted in changes in lifestyle habits and experiences of mental health outcomes, some of which were possibly related to weight gain, leading to an increase in the prevalence of obesity, which is associated with the development of several severe diseases. Concerns regarding weight gain and its impact on health outcomes are prevalent worldwide, with obesity being one of the highest causes of mortality in current society.

**Methods:**

A self-reported questionnaire collected data from participants aged 18 years of age and above from 26 countries and regions worldwide. Post-hoc multiple logistic regression analyses have been done to evaluate the association between demographic and socioeconomic factors, and the perspectives that were identified to be associated with weight gain.

**Results:**

Participants belonging to a younger age group; with a higher level of education; living in an urban area; living with family members; employed full-time; and had obesity were found to be more vulnerable to weight gain. After adjusting for socio-demographic factors, participants who were quarantined; exercised less prior to the pandemic; consumed unhealthy foods; and reported negative thoughts such as helplessness and the perceived risk of COVID-19, were more likely to experience weight gain; while negative thoughts such as having no means of control over the COVID-19 pandemic and the consequences of the COVID-19 pandemic will have great personal effect were associated with females, students, and people living in the rural area.

**Conclusions:**

Weight gain risk during the pandemic was significantly associated with certain socio-demographic and COVID-19 related factors. To improve public health outcomes, future research should conduct a longitudinal evaluation on the effects of COVID-19 experiences upon health choices. Streamlined mental support should also be provided to the vulnerable groups which were prone to negative thoughts that were associated with weight gain.

## Introduction

Since the start of the pandemic in December 2019, coronavirus disease 2019 (COVID-19) has impacted the world with more than 530 million confirmed cases and approximately 6.2 million related deaths reported from more than 220 countries and regions worldwide, as of May 2022 [1], leading to a sharp increase in the global burden and disruption of healthcare services. The COVID-19 pandemic has also caused a heightened level of social isolation, economic uncertainty, negative lifestyle habits [2], and poor mental health outcomes [3]. A recent study conducted through ecological momentary assessment (EMA) found that the pandemic had a moderate impact on students’ body weight, with an average score of 3.3 (SD = 1.0) on a five-point Likert scale [4]. It is also shown that the pandemic had a more significant effect on sedentary behavior (score: 4.3), stress levels (4.0), and depression (4.0) among students [4]. Findings suggested that the prevalence of depression symptom in the US was 3 times higher during COVID-19 than before the pandemic [5]. Also, a study in Hong Kong found that more than a quarter of the respondents reported deteriorated mental health [6], implying there is a potential rise in the prevalence of obesity as mental illnesses, such as depression, are often associated with obesity [7]. The disruption of daily life caused by the pandemic may have influenced our dietary patterns, as evidenced by a study of pregnant women who reported poorer consumption of vegetables, fruit, legumes, and dairy products compared to pre-pandemic levels [8]. However, the impact of the COVID-19 pandemic on dietary habits is still under debate [9]. The possible change in dietary patterns may be attributed to factors such as disrupted access to food, food shortages, and increased prices during the pandemic [8]. With the increased consumption of unhealthy food and decreased level of physical activity [10, 11], a long-term imbalance between energy intake and energy expenditure may often lead to weight gain and thus, the development of obesity [12, 13] Obesity is closely linked to the development of several severe diseases, hospitalization, and mortality [14–16]. It is estimated that the life expectancy for obese people could be reduced by 5 to 10 years, and the hazard ratio (HR) of all-cause mortality is significantly increased with BMI (BMI 25.0 to <30.0: HR = 1.11; 30.0 to <35.0: 1.44; 35.0 to <40.0: 1.92) [14].

Previous studies have evaluated the impact of COVID-19 on individuals with obesity and its effect on weight gain [17, 18]. However, these studies had relatively smaller sample sizes [19] or are limited in geographic scope [20, 21]. Some studies have included sub-group analyses on individuals with varying demographic and socio-economic characteristics [22], yet few have explored the impact of chronic health conditions and lifestyle factors. Additionally, some studies worked on the association between obesity and various factors, but did not capture the change in risk during COVID-19 pandemic [23]. The present study seeks to fill this gap by examining the risk factors associated with weight gain during the pandemic. It is hypothesised that socio-demographic variables, the presence of chronic health conditions (physical and mental health illnesses and conditions) and COVID-19 related experiences, behaviours, and mental health outcomes (perceptions) would impact weight change, specifically leading to increased weight gain during the pandemic. The identification of sub-groups at higher risk of weight gain will help facilitate future policy-making and the development of interventions tailored specifically for vulnerable individuals in the population.

## Methods

### Study setting

The Association of Pacific Rim Universities (APRU) Global Health Program conducted a global survey utilising an online self-report questionnaire hosted across 26 sites. Study sites include countries and regions from (1) the Asia-Pacific region: Australia, mainland China, Hong Kong, India, Indonesia, Japan, Malaysia, New Zealand, the Philippines, Russia, South Korea, Taiwan and Thailand; (2) North and Southern America: Canada, Central America, Colombia, Ecuador, Mexico, Peru and the USA; (3) Europe: The United Kingdom, France, Germany, and Italy; and (4) the Middle East: Iraq; Saudi Arabia, and Oman. The full list on the number of respondents from each of the study sites can be found in the **S1 Table in S1 File**. APRU is made up of 60 leading universities of the Pacific Rim that have received worldwide recognition for their academic excellence and contributions to research. Launched in 2007–2008, the APRU Global Health Program is an initiative that aims to address global and regional health issues through collaborative research efforts. It covers a variety of academic disciplines and topics, including non-communicable diseases.

### Recruitment

From October 2020 to December 2021 surveys were circulated online to the general population of each respective country or region through different channels, such as social media platforms and links to the website sent through emails. Study participants were recruited through the investigators’ social networks using snowball sampling, in which participants were asked to invite their friends, colleagues, classmates and relatives to complete the questionnaire. Individuals were eligible to participate if they were aged 18 years old or above, capable of comprehending the study, and able to provide informed consent. Data was sent through a submission link and stored in a secure online platform. The database was password encrypted and only accessible by research personnel to ensure confidentiality of the collected information.

### Ethical considerations

The study was approved by the Survey and Behavioural Research Ethics Committee of the Chinese University of Hong Kong (SBRE-20- 035) with ethics clearance obtained for all the study sites. Informed written consents were obtained from all respondents at the beginning of the survey.

### Survey instruments

The survey was pilot-tested and validated by an expert panel consisting of primary care professionals, epidemiologists, and physicians. The survey was available in eight languages and evaluated the association between one’s self-reported change in weight and socio-demographic variables, presence of chronic health conditions, COVID-19 related experiences, unhealthy behaviours, and mental health outcomes.

A total of 25 questions were included in the survey. All questions were optional except for the consent part (Questions 1 to 3) (where respondents were asked to confirm that they were at least 18 years old, have read the information about the study, voluntarily agreed to take part, and that they could stop the survey at any time without penalty), the date of survey, and that they have never filled out the survey.

In questions 4–11, respondents were asked about their demographic and socioeconomic status, including age, sex, race, years of education, country/ type of residence, current household composition, and work/ study status. Questions 12 was on the self-reported change in weight over the past six months using a 4-point scale (4 = weight increase, 3 = weight decrease, 2 = unchanged, 1 = unknown); while questions 13–14 were about the presence of chronic health conditions including hypertension, obesity (body mass index of 30 or above), and other conditions [cardiovascular disease (examples: coronary heart disease, heart failure, cardiomyopathy), type 2 diabetes, immunodeficiency (or taking medication, such as corticosteroid, that suppresses the immune system), chronic disease of the respiratory system (examples: asthma and chronic bronchitis), chronic liver disease, chronic kidney disease, cancer during past five years, sickle cell disease] that were grouped for analysis due to smaller number of responses, and presence of mental illnesses and conditions diagnosed by doctors or therapists self-reported by participants: depression, mania/bipolar disorder, psychotic disorders (example: schizophrenia, anxiety disorder, post-traumatic stress disorder (PTSD), eating disorder, obsessive compulsive disorder (OCD), substance use disorder (SUD), attention-deficit hyperactivity disorder (ADHD), somatoform disorder, personality disorder, autism spectrum disorder, cognitive disorder or dementia). From question 15 to 20, respondents were asked about their COVID-19 experiences: tested/ tested positive (for SARS- CoV-2 and Anti-SARS-CoV-2 antibodies), having direct contact with COVID-19 patients, experiencing COVID-19 symptoms, and quarantining. Question 21 inquired respondents about negative modifications to lifestyle following the COVID-19 pandemic: the increased consumption of alcohol, cigarettes and unhealthy food, reduction in physical exercise level, and postponement of vaccination over the past 14 days, using a Likert scale, with responses including ‘“strongly agree,” “agree,” “neutral,” “disagree,” and “strongly disagree”. Question 22 was about behaviours related to personal hygiene and social distancing in the past 14 days: tendency to not wear a mask in public, touching their eyes, nose and mouth with unwashed hands, avoiding social events, work, or school, using disinfectants and sanitiser, and washing hands frequently and properly, using the same Likert scale. Questions 23–25 asked respondents about their recent mental health: Question 23 was about the experience of negative feelings caused by media hype, helplessness, stress, fear, disinterest in daily life (Likert scale); question 24 evaluated respondents’ current perceived risk of the COVID-19 pandemic, including worries of becoming infected, dying of COVID-19, and infecting others who are close to them (Likert scale); while question 25 required respondents to indicate their general well-being over the past 14 days, sub-questions included the feeling of cheerful, calm, active, interested with daily life, and refreshed after sleep, whilst options included “all of the time”, “most of the time”, “more than half the time”, “less than half the time”, “some of the time”, and “at no time”. The survey is included in the **S1 Material in S1 File** for detailed information on the question types.

### Statistical analysis

Data were entered and statistical analyses conducted using IBM Statistical Package for Social Sciences (SPSS) version 25 software. A descriptive analysis of study participants was performed based on their demographic details and socio-economic status. Chi-square tests were deployed to compare the difference in proportion of participants experiencing weight gain across various groups. The association between factors and the experience of weight gain was examined, with a change in weight as the outcome variable and the demographic and socio-economic factors as the explanatory variables (Table 1). For analytical purposes, weight gain referred to people who reported an increase in weight over the past 6 months, while no weight gain referred to people whose weight has decreased, remained the same, or who did not know if there was any weight change. To evaluate the associations between dependent variable, weight gain, and various factors, multiple logistic regression was conducted using the enter method, in which all the variables were included in the model. It is used to investigate the presence and the strength (if any) of associations for all the independent variables. All *p* values less than 0.05 were considered statistically significant. In Table 2, the first analysis used weight change as an outcome variable, while demographic factors, socioeconomic status, presence of physical and mental illnesses/conditions (questions 4–11, 13–14) were entered as potential explanatory variables. In Table 3, the second analysis explored the association between COVID-19 related experiences (questions 15–20, as explanatory variables) and weight change (as outcome variable), with demographic factors, socioeconomic status, presence of physical and mental illnesses/conditions (questions 4–11, 13–14) entered as confounding factors. In Tables 4 and 5, the third and fourth analysis evaluated the association between health behaviours (questions 21–22, as explanatory variables) and weight change (as outcome variable), and the association between mental health outcomes (questions 23–25, as explanatory variables) and weight change (as outcome variable), respectively, using the same confounding factors. For the ease of analysis, responses to questions about lifestyle habits, behaviours related to disease prevention and mental health outcomes (questions 21–24) are coded as follows: ‘Strongly disagree’, ‘disagree’, ‘neutral’ are coded as ‘no’, while ‘agree’ and ‘strongly agree’ are coded as ‘yes’. For questions about general well-being (question 25), ‘all of the time’, ‘most of the time’, and ‘more than half of the time’ are coded as ‘yes’, while ‘less than half the time’, ‘some of the time’ and ‘at no time’ are coded as ‘no’.

**Table 1:** Characteristics of the participants.

Variables		All subjects	Weight gain = Yes	Weight gain = No	*P* *
		(N = 2553)	(N = 1087)	(N = 1466)	
**Age years** (mean ± sd)		29.33 ± 11.10	28.35 ± 9.91	30.07 ± 11.87	< .001
[median, IQR] (Range)		[25, 13] (67)	[25, 11] (57)	[26, 14] (62)	
**Sex n (row %)**					
	Male	918	374 (40.7%)	544 (59.3%)	.16
	Female	1607	701 (43.6%)	906 (56.4%)	
**Race**					
	Asian	1635	702 (42.9%)	933 (57.1%)	.36
	White	252	113 (44.8%)	139 (55.2%)	
	Black	188	68 (36.2%)	120 (63.8%)	
	American Indian or Alaska Native	97	38 (39.2%)	59 (60.8%)	
	Others	376	163 (43.4%)	213 (56.6%)	
**Years of Education**					
	0–9 years	102	35 (34.3%)	67 (65.7%)	.10
	10–12 years	360	143 (39.7%)	217 (60.3%)	
	> 12 years	2085	906 (43.5%)	1179 (56.5%)	
**Residence**					
	Urban area	1633	681 (41.7%)	952 (58.3%)	.43
	Rural area	631	277 (43.9%)	354 (56.1%)	
	Rural–urban fringe	234	106 (45.3%)	128 (54.7%)	
**Current household composition**					
	live alone	273	97 (35.5%)	176 (64.5%)	.03
	live with family	2059	901 (43.8%)	1158 (56.2%)	
	live with other people	196	80 (40.8%)	116 (59.2%)	
**Work/study status**					
	Full-time employed	1089	487 (44.7%)	602 (55.3%)	.004
	Part-time/self employed	245	85 (34.7%)	160 (65.3%)	
	Students	1025	449 (43.8%)	576 (56.2%)	
	Others^	155	53 (34.2%)	102 (65.8%)	
**Chronic physical conditions**					
	Obesity				< .001
	Yes	295	173 (58.6%)	122 (41.4%)	
	No	2175	887 (40.8%)	1288 (59.2%)	
	Hypertension				.12
	Yes	241	115 (47.7%)	126 (52.3%)	
	No	2230	948 (42.5%)	1282 (57.5%)	
	Other conditions				.001
	Yes	724	345 (47.7%)	379 (52.3%)	
	No	1829	742 (40.6%)	1087 (59.4%)	
**Mental illnesses or conditions**					
	Yes	486	194 (39.9%)	292 (60.1%)	.19
	No	2067	893 (43.2%)	1174 (56.8%)	

* Chi-square tests were used to compare the difference in proportion of participants experiencing weight gain across groups; significant at *p* < 0.05

^Including: Not employed but not student, retired, caregiver, and other

**Table 2:** Socio-demographic factors associated with increased weight in the past six months.

Variables		Multiple Logistic Regression analysis aOR (95%CI)	*P*	Stepwise Logistic Regression analysis aOR (95%CI)	*P* *
**Age years**		0.97 (0.96–0.98)	< .001	0.97 (0.96–0.98)	< .001
**Sex**					
	Male (ref)	1 (ref)			
	Female	1.16 (0.96–1.39)	.13		
**Race**					
	Asian (ref)	1 (ref)			
	White	1.28 (0.93–1.76)	.13		
	Black	0.83 (0.58–1.19)	.32		
	American Indian or Alaska Native	0.85 (0.54–1.34)	.49		
	Others	1.06 (0.82–1.37)	.66		
**Years of Education**					
	0–9 years (ref)	1 (ref)		1 (ref)	
	10–12 years	1.50 (0.88–2.54)	.13	1.57 (0.93–2.65)	.09
	> 12 years	1.84 (1.13–3.01)	.02	1.90 (1.17–3.09)	.01
**Residence**					
	Urban area (ref)	1 (ref)			
	Rural area	1.09 (0.89–1.34)	.41		
	Rural–urban fringe	1.23 (0.91–1.65)	.18		
**Current household composition**					
	live alone (ref)	1 (ref)		1 (ref)	
	live with family	1.34 (1.00–1.79)	.05	1.38 (1.04–1.84)	.03
	live with other people	1.09 (0.72–1.64)	.69	1.11 (0.74–1.67)	.62
**Work/study status**					
	Full-time (ref)	1 (ref)		1 (ref)	
	Part-time/self employed	0.59 (0.43–0.82)	.001	0.57 (0.42–0.78)	< .001
	Students	0.71 (0.56–0.90)	.005	0.71 (0.57–0.89)	.003
	Others	0.66 (0.44–0.98)	.04	0.66 (0.44–0.98)	.04
**Chronic physical conditions**					
	Obesity				
	Yes	2.58 (1.86–3.58)	< .001	2.73 (2.08–3.59)	< .001
	No	1 (ref)		1 (ref)	
	Hypertension				
	Yes	1.22 (0.86–1.73)	.28		
	No	1 (ref)			
	Others				
	Yes	1.07 (0.83–1.39)	.60		
	No	1 (ref)			
**Mental illnesses or conditions**					
	Yes	0.82 (0.65–1.05)	.11		
	No (ref)	1 (ref)			

*Significant at *p* < 0.05

**Table 3:** Associations between COVID-19 related personal experience and increased weight.

		Weight gain = Yes	Weight gain = No	Multiple and step-wise logistic regression analysis^a^	*P* *
• **Do you currently suffer from COVID-19 symptoms such as fever, dry cough, breathing problems, sore throat, loss of smell/taste, headaches, or diarrhea?**					
	Yes	85 (7.8%)	111 (7.6%)	aOR^1^ (95%CI) 0.95 (0.68–1.33)	.77
	No (ref)	1001 (92.2%)	1344 (92.4%)		
• **Have you taken any test for SARS-CoV-2?**					
	Yes	620 (57.1%)	807 (55.7%)	aOR^1^ (95%CI) 1.02 (0.85–1.22)	.84
	No	465 (42.9%)	641 (44.3%)		
• **Have you been tested positive for SARS-CoV-2?**					
	Yes	121 (18.3%)	148 (16.8%)	aOR^1^ (95%CI) 1.02 (0.76–1.39)	.88
	No	542 (81.7%)	734 (83.2%)		
• **Have you been tested positive for Anti-SARS-CoV-2 antibodies?**^**b**^					
	Yes	117 (18.3%)	146 (17.1%)	aOR^1^ (95%CI) 1.00 (0.73–1.36)	.99
	No	521 (81.7%)	709 (82.9%)		
• **Has anyone with whom you have had direct contact in the past two weeks become infected with COVID-19 that you are aware of?**					
	Yes	139 (12.9%)	211 (14.6%)	aOR^1^ (95%CI) 0.74 (0.57–0.98)	.03
	No	938 (87.1%)	1230 (85.4%)		
• **Please indicate whether you have been in quarantine due to COVID-19.**					
	Yes	235 (21.9%)	248 (17.2%)	aOR^1^ (95%CI) 1.26 (1.02–1.57)	.03
	No	837 (78.1%)	1192 (82.8%)		

^a^Adjusted for all socio-demographic factors, presence of chronic physical conditions and mental illnesses or conditions

^b^Anti-SARS-CoV-2 antibodies test is not widely available in all countries

aOR^1^ Adjusted odd ratios derived from multiple logistic regression

aOR^2^ Adjusted odd ratios derived from stepwise logistic regression

*Significant at *p* < 0.05

**Table 4:** Associations between COVID-19 related health behaviours and increased weight.

		Weight gain = Yes	Weight gain = No	Multiple and stepwise logistic regression analysis^a^	*P* *
• **Have consumed substantially more alcohol than usual.**					
	Yes	120 (11.5%)	141 (10.0%)	aOR^1^ (95%CI) 1.18 (0.87–1.58)	
	No	923 (88.5%)	1270 (90.0%)		.29
• **Have smoked considerably more cigarettes than usual.**					
	Yes	82 (7.8%)	115 (8.2%)	aOR^1^ (95%CI) 1.01 (0.70–1.47)	.96
	No	964 (92.2%)	1294 (91.8%)	aOR^2^ (95%CI) 0.72 (0.51–1.01)	.06
• **Exercised less than I did before the pandemic.**					
	Yes	442 (42.3%)	430 (30.6%)	aOR^1^ (95%CI) 1.65 (1.37–1.97)	< .001
	No	602 (57.7%)	977 (69.4%)	aOR^2^ (95%CI) 1.34 (1.10–1.62)	.004
• **Ate more unhealthy food than I did before the pandemic (such as fried food, coke, etc).**					
	Yes	409 (39.1%)	288 (20.5%)	aOR^1^ (95%CI) 2.46 (2.02–2.99)	< .001
	No	636 (60.9%)	1120 (79.5%)	aOR^2^ (95%CI) 2.39 (1.93–2.95)	< .001
• **Postponed vaccination for myself or my child.**					
	Yes	138 (13.3%)	163 (11.6%)	aOR^1^ (95%CI) 1.16 (0.88–1.52)	.31
	No	902 (86.7%)	1246 (88.4%)		
• **Frequently washed my hands with soap and water for at least 20 seconds.**					
	Yes	810 (76.8%)	1047 (74.0%)	aOR^1^ (95%CI) 1.12 (0.91–1.37)	.28
	No	245 (23.2%)	367 (26.0%)		
• **Avoided touching my eyes, nose and mouth with unwashed hands.**					
	Yes	767 (72.7%)	983 (69.6%)	aOR^1^ (95%CI) 1.16 (0.95–1.41)	.14
	No	288 (27.3%)	430 (30.4%)		
• **Used disinfectants/sanitizer to clean hands when soap and water were not available.**					
	Yes	838 (79.5%)	1074 (76.1%)	aOR^1^ (95%CI) 1.21 (0.98–1.50)	.07
	No	216 (20.5%)	337 (23.9%)		
• **Avoided a social event I wanted to attend.**					
	Yes	703 (66.8%)	913 (64.7%)	aOR^1^ (95%CI) 1.04 (0.87–1.26)	.65
	No	349 (33.2%)	498 (35.3%)		
• **Stayed at home from work/school.**					
	Yes	632 (60.1%)	832 (59.0%)	aOR^1^ (95%CI) 1.05 (0.88–1.26)	.58
	No	420 (39.9%)	579 (41.0%)		
• **Wore a mask in public.**					
	Yes	969 (91.8%)	1273 (90.2%)	aOR^1^ (95%CI) 1.11 (0.81–1.53)	.51
	No	87 (8.2%)	139 (9.8%)		
• **Ensured physical distancing in public**.					
	Yes	896 (85.3%)	1187 (84.2%)	aOR^1^ (95%CI) 1.06 (0.82–1.35)	.67
	No	155 (14.7%)	223 (15.8%)		
• **Disinfected surfaces.**					
	Yes	749 (70.9%)	957 (67.7%)	aOR^1^ (95%CI) 1.11 (0.92–1.34)	.29
	No	307 (29.1%)	456 (32.3%)		

^a^Adjusted for all socio-demographic factors, presence of chronic physical conditions and mental illnesses or conditions

aOR^1^ Adjusted odd ratios derived from multiple logistic regression

aOR^2^ Adjusted odd ratios derived from stepwise logistic regression

*Significant at *p* < 0.05

**Table 5:** Associations between COVID-19 related mental health outcomes and weight change.

		Weight gain = Yes	Weight gain = No	Simple and multiple logistic regression analysis^a^	*P* *
• **COVID-19 to me feels spreading slowly**					
	Yes	373 (36.3%)	518 (37.5%)	aOR^1^ (95%CI) 0.86 (0.71–1.04)	.11
	No	654 (63.7%)	862 (62.5%)	aOR^2^ (95%CI) 0.85 (0.71–1.02)	.08
• **COVID-19 to me feels something I think about all the time**					
	Yes	437 (42.7%)	550 (39.6%)	aOR^1^ (95%CI) 1.11 (0.93–1.32)	.25
	No	587 (57.3%)	838 (60.4%)		
• **COVID-19 to me feels fear-inducing**					
	Yes	562 (54.9%)	762 (55.0%)	aOR^1^ (95%CI) 0.98 (0.82–1.16)	.79
	No	462 (45.1%)	624 (45.0%)		
• **COVID-19 to me feels media hyped**					
	Yes	495 (48.3%)	664 (48.1%)	aOR^1^ (95%CI) 0.96 (0.80–1.16)	.67
	No	530 (51.7%)	716 (51.9%)		
• **COVID-19 to me feels something that makes me feel helpless**					
	Yes	410 (40.0%)	581 (41.9%)	aOR^1^ (95%CI) 0.90 (0.76–1.08)	.26
	No	614 (60.0%)	805 (58.1%)		
• **COVID-19 to me feels highly stressful**					
	Yes	569 (55.4%)	742 (53.5%)	aOR^1^ (95%CI) 1.04 (0.88–1.24)	.64
	No	458 (44.6%)	645 (46.5%)		
• **I have no means of control over the COVID-19 pandemic**					
	Yes	516 (50.3%)	624 (45.0%)	aOR^1^ (95%CI) 1.20 (1.01–1.43)	.04
	No	510 (49.7%)	763 (55.0%)		
• **I will become infected with COVID-19**					
	Yes	468 (45.5%)	541 (39.0%)	aOR^1^ (95%CI) 1.18 (0.99–1.41)	.07
	No	560 (54.5%)	845 (61.0%)		
• **People close to me are going to be infected with COVID-19**					
	Yes	537 (52.3%)	653 (47.0%)	aOR^1^ (95%CI) 1.13 (0.95–1.35)	.17
	No	489 (47.7%)	735 (53.0%)		
• **The consequences of the COVID-19 pandemic will greatly affect me personally**					
	Yes	590 (57.4%)	708 (51.2%)	aOR^1^ (95%CI) 1.28 (1.07–1.52)	.007
	No	437 (42.6%)	676 (48.8%)	aOR^2^ (95%CI) 1.30 (1.08–1.55)	.004
• **I will die of COVID-19**					
	Yes	267 (26.0%)	302 (21.8%)	aOR^1^ (95%CI) 1.18 (0.96–1.45)	.12
	No	759 (74.0%)	1084 (78.2%)		
• **I have felt cheerful and in good spirits**					
	Yes	672 (66.3%)	952 (68.9%)	aOR^1^ (95%CI) 0.94 (0.78–1.14)	.56
	No	341 (33.7%)	430 (31.1%)		
• **I have felt calm and relaxed**					
	Yes	636 (62.8%)	891 (64.9%)	aOR^1^ (95%CI) 1.00 (0.83–1.20)	.98
	No	376 (37.2%)	481 (35.1%)		
• **I have felt active and vigorous**					
	Yes	606 (60.2%)	863 (62.8%)	aOR^1^ (95%CI) 0.96 (0.80–1.16)	.70
	No	401 (39.8%)	511 (37.2%)		
• **I have woken up feeling fresh and rested**					
	Yes	551 (54.7%)	767 (55.9%)	aOR^1^ (95%CI) 1.04 (0.87–1.25)	.66
	No	456 (45.3%)	605 (44.1%)		
• **My daily life has been filled with things that interest me**					
	Yes	608 (60.1%)	851 (61.7%)	aOR^1^ (95%CI) 1.04 (0.86–1.24)	.70
	No	404 (39.9%)	529 (38.3%)		

^a^Adjusted for all socio-demographic factors, presence of chronic physical conditions and mental illnesses or conditions

aOR^1^ Adjusted odd ratios derived from multiple logistic regression

aOR^2^ Adjusted odd ratios derived from stepwise logistic regression

*Significant at *p* < 0.01

Additional separate backward stepwise logistic regression models were set up to access COVID-19 related experiences, unhealthy behaviours, and mental health outcomes separately as predictors of weight change, whilst the potential confounding socio-demographic factors were controlled for. At each step, variables were chosen based on p-values: the variable having the lowest correlation will be removed from the model if it has a p-value of 0.1 or above. The procedure ends when no variables left in the model satisfy the elimination criterion. In the study, only complete datasets are included for analysis. To check for correlations among independent variables, multicollinearity was examined using the variance inflation factor (VIF) with a cut-off at 5. Heat maps and tree-like hierarchal clustering lines were used to present the correlation among the confounding factors. Post-hoc multiple logistic regression was conducted on perspectives that were associated with weight gain to identify high risk groups vulnerable to developing negative mental health outcomes and to analyse the effect of regions of responses.

## Results

### Participant characteristics

A total of 2,553 surveys were collected with 295 (11.6% of all participants) individuals with obesity (body mass index ≥ 30 kg/m^2^) and 241 (9.4%) participants having hypertension. These two were the most prevalent conditions reported amongst participants with any type of chronic physical conditions (N = 724; 28.4%). A total of 42.6% of respondents reported an increase in weight (N = 1087) over the past six months, whilst 14.1% and 38.6% reported weight loss (N = 359) and no change in weight (N = 986), respectively. Approximately one-fifth of the respondents (N = 486; 19.0%) reported the presence of at least one form of mental illness or conditions, such as depression or anxiety. Respondents had a mean age of 29.33 with a standard deviation of 11.10 years, the majority were female (N = 1607; 62.9%). More than half of the respondents were Asian (N = 1635, 64.0%), had more than 12 years of education (N = 2085; 81.7%), living in urban area (N = 1633; 64.0%), and living with family members (N = 2059; 80.7%). In terms of socio-economic status, approximately 42.7% were working full-time (N = 1089) and 40.1% were students (N = 1025). More than half of the responses were collected between September and October 2021 (N = 1352; 53.0%). Detailed demographic and socio-economic statistics of the participants are listed in **Table 1**; the tables of distribution of responses over time by country are listed in **S2b Table in S1 File**.

### Socio-demographic factors associated with increased weight in the past six months

Several factors were found to be associated with an increase in weight **(Table 2)**. Age was negatively associated with weight gain, after adjusting for other factors (aOR = 0.97, 95% CI: 0.96–0.98, *P* < .001), with the younger population being more prone to weight gain. In the survey, respondents reporting an increase in weight over the past six months had a mean age of 28.35 (sd = 9.91), whilst those who did not gain weight had a mean age of 30.07 (sd = 11.87). Individuals with more than 12 years of education had a higher risk of weight gain than people who had nine years of education or less (43.5% vs 34.3%; aOR = 1.84, 95% CI: 1.13–3.01; *P* = .02). Respondents living with family (43.8%; aOR = 1.34, 95% CI: 1.00–1.79, *P* = .048) were at greater risk of experiencing a weight gain compared to respondents living alone, with 35.5% of them having experienced a weight gain). Furthermore, respondents of different forms of employment had significantly different experiences in weight gain: Compared to respondents working full time (44.7%), respondents working part-time or who were self-employed (34.7%; aOR = 0.59, 95% CI: 0.43–0.82, *P* = .001), students (43.8%; aOR = 0.71, 95% CI: 0.56–0.90, *P* = .005), and others (retired, unemployed, etc.) (34.2%; aOR = 0.66, 95% CI: 0.44–0.98, *P* = .04) were all less likely to experience an increase in weight. Respondents with obesity were found to be more likely to gain weight (58.6% versus 40.8% who were not; aOR = 2.58, 95% CI: 1.86–3.58, *P* < .001).

### Associations between COVID-19 experiences, behaviours, mental health and weight gain

After adjusting for socio-demographic factors, individuals who have had direct contact with people who were infected with COVID-19 in the previous two weeks (12.9% vs 14.6%; aOR = 0.74, 95% CI: 0.57–0.98, *P* = .03) were less likely to experience weight gain over the past six months. Conversely, people who had been quarantined due to COVID-19 (21.9% vs 17.2%; aOR = 1.26, 95% CI: 1.02–1.57, *P* = .03) were more likely to have gained weight in the multiple logistic regression model **(Table 3)**. In stepwise logistic regression, all of the variables were insignificant and eliminated in the process.

As far as behavioural habits were concerned, it was found that individuals who exercised less now compared to before the pandemic (42.3% versus 30.6%; aOR = 1.65, 95% CI: 1.37–1.97; *P* < .001) and ate more unhealthy food such as deep-fried foods and fizzy drinks (39.1% versus 20.5%; aOR = 2.46, 95% CI: 2.02–2.99; *P* < .001) were significantly more likely to gain weight (**Table 4**). In the backward stepwise logistic regression, similar findings were observed as exercising less and eating more unhealthy food were identified as risk factors of gaining weight (**Table 4**).

People who had negative thoughts regarding COVID-19 outcomes, including feeling like they had no means of control over the pandemic (50.3% vs 45.0%; aOR = 1.20, 95% CI: 1.01–1.43; *P* = .04), and the consequences of the pandemic would greatly affect them personally (57.4% vs 51.2%; aOR = 1.28, 95% CI: 1.07–1.52; *P* = .007) had a tendency to experience weight gain after adjusting for demographic variables (**Table 5**). Likewise, the backward stepwise logistic regression model identified the thought that the consequences of the pandemic would greatly affect them personally as potential risk factors for experiencing a weight gain **(Table 5).**

Multicollinearity was not detected among the variables as all of them had VIF below 5, the detailed VIFs for the variables are listed in **S3 Table in S1 File.**

Furthermore, heat maps and tree-like hierarchical clustering lines were used to show the correlation including all the confounding factors. It was found that age was more related to chronic conditions than other demographic factors, while education level and current household composition were more related to media hyped and COVID-19 spreading (**S5 Table in S1 File**). Details regarding the clustering effects for other factors can be found in **S5 Table in S1 File.**

Sensitivity analysis has been done by including the regions of responses into the multivariable logistic regression model. It was found that the demographic and socioeconomic factors that were significant with weight gain were consistent with the original model. The detailed results are listed in **S6 Table in S1 File.**

## Discussion

### Principal results

The aim of the study was to evaluate factors that heightened the likelihood of weight gain during the COVID-19 pandemic. We analysed demographic background, socio-economic status, pre-existing chronic health conditions and COVID-19 related experiences, behaviours and negative mental thoughts. The association analysis indicated that younger age, longer years of education, current household composition (with family members living together), forms of employment (full-time workers), and obesity, were associated with a higher risk of weight gain. The hypothesis was further supported as the following factors were associated with an increase in weight during the COVID-19 outbreak: (1) individuals who had undergone quarantine due to COVID-19; (2) participants who developed unhealthy habits following the pandemic, such as exercising less and consuming more unhealthy foods compared to before; (3) negative thoughts such as, feelings of helplessness in relation to the pandemic, pessimism towards becoming infected with COVID-19, passing away due to COVID-19, or concern of close contacts contracting COVID-19.

### Limitations

The current study had a few limitations. Firstly, the response rate of the survey could not be evaluated as the usage of a consecutive sampling strategy means that the number of participants who received the survey invitation is unknown. Additionally, the survey responses were submitted over a lengthy interval of time of approximately one year and two months. The incidence and mortality rate of COVID-19 cases fluctuated with time; therefore, it is difficult to evaluate how or whether the severity of the pandemic, public health measures introduced by the government, and differing forms of social distancing policies implemented during various waves of COVID-19 would impact the outcome variables. As such, associations of the risk factors with weight gain may be heterogenous across study sites due to differences in the spread of COVID-19 and related policies. As change in weight was recorded using self-report measures, there is the possibility of inaccurate responses due to recall bias. Also, the nutritional status of the respondents may not be fully reflected, as the change in weight was used as the sole measurement for the outcome, since other indicators such as subcutaneous fold thickness, lean body mass, and calf circumference were difficult to be included in a self-reported questionnaire. Furthermore, generalising the findings of this study for other settings should be done with caution, as data was collected using a non-random sampling strategy and not all countries provided a large number of responses. Selection bias may be present since the online setting of the current survey might have had a lower chance of attracting older participants who are not regular internet users or do not have internet access, future studies can be done in an offline setting to explore the association among the older population. Moreover, despite the establishment of an association between the increased weight and mental health outcomes and behavioural changes, the study did not evaluate the potential causes underlying the relationship. Some possible confounding factors, including the extent and duration of the lockdown policies across different countries, availability of mental health support, mask mandates, and variants of COVID-19 were not included in the questionnaire. As a result, further exploration on detailed pathways and potential confounders may be required to determine how recent weight gain might influence personal behaviours and mental health overall.

### Explanations and comparisons with existing literature

Certain socio-demographic factors have been associated with a higher likelihood of weight gaining which consequently may increase the prevalence of medical conditions, such as obesity. Individuals with obesity often experience respiratory dysfunction, compromised metabolic health, and are predisposed to diseases associated with pneumonia-related organ failure. This increase their risk of suffering adverse health outcomes from severe COVID-19 [24].

In line with previous studies, our study found that younger individuals were more prone to weight gain compared to the older population during the pandemic, possibly because age-related reductions in appetite and energy intake [25]. Moreover, older adults were shown to be more resilient in the face of pandemic-induced stressors, and were less likely to adopt detrimental habits related to sleep, diet, and physical inactivity [26]. Our study also revealed an inverse association between age and stress (aOR: 0.99, 95% CI: 0.98–1.00, *P* = .04, **S4d Table in S1 File**), indicating that older people tended to experience less stress. It is possible that age confounded the variable of stress in the analysis for weight gain.

In the current study, it was shown that individuals with a higher level of educational attainment (> 12 years) were more at risk of weight gain than those who were less educated (< 9 years). This finding contradicts the prevailing perspective in the existing literature, which suggest that individual with tertiary education are less vulnerable to weight gain due to greater nutritional knowledge and increased access to healthier dietary options [27–29]. People with higher education levels are more likely to engage in white-collar jobs, which is characterised by prolonged sitting time. In the context of the pandemic, the closure of gyms, limited outdoor activities, and work-from-home arrangements may have a greater impact on white-collar workers in terms of physical activity, as they may be more prone to a sedentary lifestyle due to their work nature. A previous study reported a significant reduction in physical activity (Total: –0.41%, p<0.001; Sport-related: -0.52%, p<0.001) when working from home among 160 office workers [30]. This may pose a greater risk of weight gain among people with higher education level. In addition, full-time employment may lead to a more sedentary lifestyle, as workers spend more time at their desks and less time engaging in physical activities. Combined with occupational stress, which can negatively affect eating patterns, and having more funds to purchase food, full-time employees are often at higher risk of weight gain [31, 32]. Our study supports these findings by demonstrating that people with all other forms of employment statuses—part-time, self-employed or unemployed—were less likely to experience weight gain compared to those in full-time employment.

Turning to physical health status, most chronic physical conditions were not found a significant relationship with weight gain. Although hypertension has been consistently associated with obesity [33], it was not identified as a significant factor in this study. It might be due to the younger age of the sample population, in which the prevalence of hypertension was 9.5%—a similar prevalence compared to studies among younger adults (USA– 7.3% [34], India– 11.2% [35] and Uganda– 15% [36]) but considerably lower than the overall prevalence of over 50% in the whole population [37]. However, the bi-directional relationship between obesity and health conditions may precipitate weight gain in a long run which was observed in this study as individuals reporting obesity were at higher risk of weight gain [33]. Other chronic physical conditions were found significant in the simple binary logistic regression. However, it was not significant in multivariable logistic regression after confounding for other demographic factors such as age, gender, and education, possibly because it was not an independent explanatory factor. Pre-existing health conditions related to obesity have also been predictive of cardiovascular disease, type 2 diabetes, musculoskeletal disorders, and almost all cancers, which can lead to premature mortality [38]. With the restrictive lifestyle changes imposed on the population due to the pandemic, the health management of those with chronic health conditions may deteriorate and consequently be more severely impacted by COVID-19.

The restrictions enforced during the pandemic had numerous consequences upon some population’s health, with COVID-19 related experiences, behavioural changes, and poor mental health outcomes brought about by negative thoughts, contributing to an increase in weight. In this study, individuals who had experienced quarantine were found to be more at risk of weight gain, reinforcing findings wherein self-isolation and social distance led to an increased desire for food, higher levels of energy intake, and insufficient sleep and exercise [39, 40]. Although behaviours related to social events, such as social distancing and avoiding social events, were not found to significantly impact the likelihood of weight gain [which was possibly due to its stronger correlation with age (aOR: 1.03, 95% CI: 1.02–1.04, *P* < .001) and residence (rural area vs urban area; aOR: 1.66, 95% CI 1.23–2.19, *P* = .001), **S4c Table in S1 File**)], it was closely related to certain health habits such as reduced physical activity and increased consumption of unhealthy foods. Individuals may frequently resort to negative lifestyle choices as coping mechanisms when faced with stressors. These choices can reinforce prior behavioural and include behaviors such as overeating, increased frequency of meals, and higher consumption of alcohol and smoking cigarettes [41, 42]. However, higher consumption of alcohol and smoking cigarettes were not significant in this study, possibly due to the difference in study design, as the study had a relatively young sample population.

Negative mental health outcomes may be an indicator of experiencing prolonged exposure to stressful situations. Inhibition of appetite and seeking out nutrient-dense foods have both been responses to stress, with the latter increasing overall energy intake [43]. As such, negative thoughts—classified as helplessness, perceived risks, concerns of becoming infected—was an indicator of a higher likelihood for increased weight. In our study, additional analyses have shown that females (aOR_no control_: 1.33, 95% CI: 1.11–1.61, *P* = .002; aOR_greatly affect_: 1.36, 95% CI: 1.13–1.64, *P* = .001), students (aOR_no control_: 1.35, 95% CI: 1.07–1.71, *P* = .012; aOR_greatly affect_: 1.54, 95% CI: 1.22–1.95, *P* < .001), people living in rural area (aOR_no control_: 1.38, 95% CI: 1.12–1.69, *P* = .002; aOR_greatly affect_: 1.42, 95% CI: 1.15–1.75, *P* = .001) were more likely to experience negative thoughts contributing to weight change (having no means of control over the COVID-19 pandemic and the consequences of the COVID-19 pandemic will greatly affect them personally), indicating the importance of streamlined interventions for these high-risk groups **(S4a, S4b Tables in S1 File)**.

### Implications

The present study has identified certain high-risk groups of individuals who are vulnerable to weight increase, including those who work full-time and individuals with obesity. The increase in weight among full-time workers may be attributed to the implementation of work-from-home policies, which may lead to significant increase in working schedules and a reduction in physical activity. The results of the group with obesity was particularly alarming since they were negatively impacted pandemic as their chronic condition worsened. It is imperative to recognise that weight gain is not a stand-alone observation, but rather an indication of the development of more severe medical conditions and an increased global burden on the healthcare system. Furthermore, individuals who had experienced mental illness or conditions were found to be more vulnerable to gaining weight. Therefore, mental health support groups and social support programmes should be considered to ameliorate the negative mental health outcomes brought by the pandemic. Advice should be offered to the general public concerning behavioural and lifestyle modifications, particularly for those who work from home. Analyses also identified females, students, and people living in the rural area as potential high-risk groups for experiencing negative thoughts that contribute to weight gain. This underscores the needs for early interventions such as targeted mental support before such thoughts turned into physical outcomes of weight gain or obesity.

## Conclusion

Several demographic and socioeconomic factors, as well as COVID-19 experiences, lifestyle modifications, and mental health outcomes, were associated with weight gain. Further research should explore alternative interventions to target various groups of high-risk individuals, whilst follow-up surveys should be done periodically to identify and track the temporal trends and to evaluate whether the impact of COVID-19 experiences and behaviours persist.

## Supporting information

S1 File Appendix (includes S1-S7 Tables & S1 Material). (DOCX)
