# Supplementary material for: Factors associated with weight gain during COVID-19 pandemic: A global study
Source: PLoS One. 2023 Apr 20;18(4):e0284283. doi: 10.1371/journal.pone.0284283 (PMC10118192; doi:10.1371/journal.pone.0284283)
Supplement: S1 File — (DOCX) [file pone.0284283.s001.docx]

**Appendix Legends**

**Supplementary Table 1** Number of responses by country

**Supplementary Table 2a** Number of responses over time (overall)

**Supplementary Table 2b** Number of responses over time by country

**Supplementary Table 3** Multicollinearity

**Supplementary Table 4a** Association between demographic factors and feeling of no control

**Supplementary Table 4b** Association between demographic factors and feeling of being affected greatly by COVID-19

**Supplementary Table 4c** Association between demographic factors and avoiding social events

**Supplementary Table 4d** Association between demographic factors and feeling highly stressful

**Supplementary Table 5a** Heat map of all factors

**Supplementary Table 5b** Tree-like hierarchical clustering lines of all factors

**Supplementary Table 6** Socio-demographic factors associated with increased weight in the past six months (sensitivity analysis with regions)

**Supplementary Table 7** Survey instruments

**Supplementary Material 1** Questionnaire

**Supplementary table 1** Number of responses by country

| **Country** | **N** |
| --- | --- |
| Philippines | 897 |
| China | 492 |
| Ecuador | 287 |
| United States | 282 |
| Mexico | 195 |
| Thailand | 193 |
| Australia | 57 |
| Canada | 45 |
| Kingdom of Saudi Arabia | 16 |
| Japan | 9 |
| South Korea | 9 |
| Malaysia | 9 |
| New Zealand | 4 |
| France | 3 |
| United Kingdom | 2 |
| Singapore | 2 |
| Switzerland | 1 |
| India | 1 |
| Colombia | 1 |
| Nigeria | 1 |
| Germany | 1 |
| Oman | 1 |
| Italy | 1 |
| Indonesia | 1 |
| Taiwan | 1 |
| Pakistan | 1 |
| United Arab Emirates | 1 |
| Peru | 1 |
| Nepal | 1 |
| Central America | 1 |
| Iraq | 1 |
| Russia | 1 |
| Missing | 35 |
| **Total** | **2553** |

**Supplementary table 2a** Number of responses over time (overall)

| Year | Month | Weight gain (N, %) | No weight gain | Total |
| --- | --- | --- | --- | --- |
| 2021 | Jan | 237, 68.3% | 110, 31.7% | 347, 13.6% |
|  | Feb | 44, 57.9% | 32, 42.1% | 76, 3.0% |
|  | Mar | 29, 65.9% | 15, 34.1% | 44, 1.7% |
|  | Apr | 37, 64.9% | 20, 35.1% | 57, 2.2% |
|  | May | 22, 59.5% | 15, 40.5% | 37, 1.4% |
|  | Jun | 85, 64.9% | 46, 35.1% | 131, 5.1% |
|  | Jul | 85, 63.9% | 48, 36.1% | 133, 5.2% |
|  | Aug | 64, 58.7% | 45, 41.3% | 109, 4.3% |
|  | Sep | 423, 54.4% | 354, 45.6% | 777, 30.4% |
|  | Oct | 311, 54.1% | 264, 45.9% | 575, 22.5% |
|  | Nov | 34, 42.5% | 46, 57.5% | 80, 3.1% |
|  | Dec | 9, 75.0% | 3, 25.0% | 12, 0.5% |
| 2022 | Jan | 86, 49.1% | 89, 50.9% | 175, 6.9% |
|  | Total | **1466** | **1087** | **2553** |

**Supplementary table 2b** Number of responses over time by country

|  |  | 202101 | 202102 | 202103 | 202104 | 202105 | 202106 | 202107 | 202108 | 202109 | 202110 | 202111 | 202112 | 202201 |
| --- | --- | --- | --- | --- | --- | --- | --- | --- | --- | --- | --- | --- | --- | --- |
| Philippines | Others | 0 | 0 | 0 | 2 | 0 | 3 | 2 | 17 | 101 | 218 | 27 | 7 | 81 |
|  | Increased weight | 0 | 1 | 0 | 0 | 0 | 2 | 0 | 19 | 106 | 189 | 39 | 3 | 80 |
|  | Total | 0 | 1 | 0 | 2 | 0 | 5 | 2 | 36 | 207 | 407 | 66 | 10 | 161 |
| China | Others | 223 | 34 | 22 | 32 | 8 | 0 | 0 | 0 | 7 | 1 | 0 | 0 | 0 |
|  | Increased weight | 106 | 21 | 13 | 15 | 6 | 0 | 0 | 0 | 4 | 0 | 0 | 0 | 0 |
|  | Total | 329 | 55 | 35 | 47 | 14 | 0 | 0 | 0 | 11 | 1 | 0 | 0 | 0 |
| Ecuador | Others | 0 | 0 | 0 | 0 | 0 | 0 | 2 | 4 | 120 | 34 | 0 | 0 | 0 |
|  | Increased weight | 0 | 0 | 0 | 0 | 0 | 0 | 0 | 3 | 98 | 25 | 1 | 0 | 0 |
|  | Total | 0 | 0 | 0 | 0 | 0 | 0 | 2 | 7 | 218 | 59 | 1 | 0 | 0 |
| United States | Others | 1 | 5 | 0 | 0 | 1 | 33 | 1 | 0 | 111 | 6 | 0 | 0 | 0 |
|  | Increased weight | 2 | 4 | 1 | 0 | 2 | 16 | 1 | 0 | 93 | 3 | 1 | 0 | 1 |
|  | Total | 3 | 9 | 1 | 0 | 3 | 49 | 2 | 0 | 204 | 9 | 1 | 0 | 1 |
| Mexico | Others | 0 | 0 | 0 | 2 | 12 | 33 | 0 | 7 | 15 | 48 | 2 | 0 | 0 |
|  | Increased weight | 0 | 0 | 0 | 0 | 6 | 23 | 0 | 4 | 5 | 38 | 0 | 0 | 0 |
|  | Total | 0 | 0 | 0 | 2 | 18 | 56 | 0 | 11 | 20 | 86 | 2 | 0 | 0 |
| Thailand | Others | 1 | 0 | 0 | 0 | 0 | 0 | 78 | 28 | 6 | 1 | 2 | 2 | 0 |
|  | Increased weight | 0 | 0 | 0 | 1 | 0 | 0 | 47 | 15 | 7 | 4 | 0 | 0 | 1 |
|  | Total | 1 | 0 | 0 | 1 | 0 | 0 | 125 | 43 | 13 | 5 | 2 | 2 | 1 |
| Others | Others | 12 | 5 | 7 | 1 | 1 | 16 | 2 | 8 | 63 | 3 | 3 | 0 | 5 |
|  | Increased weight | 2 | 6 | 1 | 4 | 1 | 5 | 0 | 4 | 41 | 5 | 5 | 0 | 7 |
|  | Total | 14 | 11 | 8 | 5 | 2 | 21 | 2 | 12 | 104 | 8 | 8 | 0 | 12 |

**Supplementary table 3** Multicollinearity

|  | VIF |
| --- | --- |
| Do you currently suffer from COVID-19 symptoms such as fever, dry cough, breathing problems, sore throat, loss of smell/taste, headaches, or diarrhea? | 1.518 |
| Have you taken any test for SARS-CoV-2? | 1.170 |
| Have you been tested positive for SARS-CoV-2? | 2.074 |
| Have you been tested positive for Anti-SARS-CoV-2 antibodies? | 2.005 |
| Has anyone with whom you have had direct contact in the past two weeks become infected with COVID-19 that you are aware of? | 1.328 |
| Please indicate whether you have been in quarantine due to Covid-19. | 1.334 |
| Have consumed substantially more alcohol than usual. | 1.511 |
| Have smoked considerably more cigarettes than usual. | 1.587 |
| Exercised less than I did before the pandemic. | 1.322 |
| Ate more unhealthy food than I did before the pandemic (such as fried food, coke, etc). | 1.433 |
| Postponed vaccination for myself or my child. | 1.270 |
| Frequently washed my hands with soap and water for at least 20 seconds. | 1.770 |
| Avoided touching my eyes, nose and mouth with unwashed hands. | 1.611 |
| Used disinfectants/sanitizer to clean hands when soap and water were not available. | 2.087 |
| Avoided a social event I wanted to attend. | 1.505 |
| Stayed at home from work/school. | 1.534 |
| Wore a mask in public. | 1.434 |
| Ensured physical distancing in public. | 1.486 |
| Disinfected surfaces. | 1.704 |
| COVID-19 to me feels spreading slowly | 1.473 |
| COVID-19 to me feels something I think about all the time | 1.461 |
| COVID-19 to me feels fear-inducing | 1.625 |
| COVID-19 to me feels media hyped | 1.507 |
| COVID-19 to me feels something that makes me feel helpless | 1.572 |
| COVID-19 to me feels highly stressful | 1.671 |
| I have no means of control over the COVID-19 pandemic | 1.448 |
| I will become infected with COVID-19 | 2.201 |
| People close to me are going to be infected with COVID-19 | 2.119 |
| The consequences of the COVID-19 pandemic will greatly affect me personally | 1.508 |
| I will die of COVID-19 | 1.672 |
| I have felt cheerful and in good spirits | 3.204 |
| I have felt calm and relaxed | 2.944 |
| I have felt active and vigorous | 3.175 |
| I have woken up feeling fresh and rested | 2.571 |
| My daily life has been filled with things that interest me | 2.275 |
| Age | 1.507 |
| Sex | 1.196 |
| Race | 1.518 |
| Years of Education | 1.206 |
| Type of Residence | 1.075 |
| Living status | 1.102 |
| Work | 1.397 |
| Presence of obesity | 1.716 |
| Presence of hypertension | 1.598 |
| Presence of other chronic physical condition | 2.304 |
| Presence of mental illnesses | 1.316 |

**Supplementary table 4a** – Association between demographic factors and feeling of no control

|  | Multiple logistic regression | |
| --- | --- | --- |
|  | aOR (95% C.I.) | *P* |
| Age (years) | 0.998 (0.988-1.009) | .77 |
| Gender |  |  |
| Male | Ref |  |
| Female | **1.333 (1.107-1.605)** | **.002*** |
| Race |  | **.01*** |
| Asian | 1 (Ref) |  |
| White | **1.762 (1.285-2.417)** | **<.001*** |
| Black | 1.034 (0.723-1.478) | .86 |
| American Indian or Alaska Native | 0.924 (0.581-1.469) | .74 |
| Others | 0.920 (0.702-1.206) | .55 |
| Working Status |  | .07 |
| Full-time employed | 1 (Ref) |  |
| Part-time/ self employed | 1.107 (0.817-1.500) | .51 |
| Student | **1.353 (1.069-1.712)** | **.01*** |
| Others | 1.297 (0.887-1.895) | .18 |
| Years of education |  | .64 |
| 0-9 years | 1 (Ref) |  |
| 10-12 years | 1.274 (0.769-2.111) | .35 |
| More than 12 years | 1.230 (0.771-1.962) | .38 |
| Residence |  | .21 |
| Urban area | 1 (Ref) |  |
| Rural area | **1.377 (1.121-1.692)** | **.002*** |
| Rural-urban fringe | 1.212 (0.899-1.633) | .21 |
| Living status |  | .09 |
| Live alone | 1 (Ref) |  |
| Live with family | 1.024 (0.772-1.358) | .87 |
| Live with other people | 0.710 (0.470-1.073) | .10 |
| Chronic physical conditions |  |  |
| Obesity |  |  |
| - Yes | 1.098 (0.794-1.519) | .57 |
| - No | 1 (Ref) |  |
| Hypertension |  |  |
| - Yes | 1.009 (0.713-1.428) | .96 |
| - No | 1 (Ref) |  |
| Other chronic conditions |  |  |
| - Yes | 1.084 (0.835-1.406) | .55 |
| - No | 1 (Ref) |  |
| Chronic mental illnesses |  |  |
| - Yes | 1.175 (0.924-1.493) | .19 |
| - No | 1 (Ref) |  |

**Supplementary table 4b** – Association between demographic factors and feeling of being affected greatly by COVID-19

|  | Multiple logistic regression | |
| --- | --- | --- |
|  | aOR (95% C.I.) | *P* |
| Age (years) | 0.999 (0.989-1.010) | .91 |
| Gender |  |  |
| Male | Ref |  |
| Female | **1.362 (1.130-1.640)** | **.001*** |
| Race |  | **.01*** |
| Asian | 1 (Ref) |  |
| White | 1.188 (0.864-1.634) | .84 |
| Black | 0.720 (0.502-1.032) | .07 |
| American Indian or Alaska Native | 0.954 (0.596-1.526) | .84 |
| Others | **0.674 (0.513-0.885)** | **.004*** |
| Working Status |  | **.004*** |
| Full-time employed | 1 (Ref) |  |
| Part-time/ self employed | 1.131 (0.834-1.534) | .43 |
| Student | **1.539 (1.215-1.949)** | **<.001*** |
| Others | 0.991 (0.676-1.453) | .96 |
| Years of education |  | .59 |
| 0-9 years | 1 (Ref) |  |
| 10-12 years | 1.295 (0.783-2.142) | .31 |
| More than 12 years | 1.257 (0.792-1.997) | .33 |
| Residence |  | **.003*** |
| Urban area | 1 (Ref) |  |
| Rural area | **1.418 (1.149-1.750)** | **.001*** |
| Rural-urban fringe | 1.285 (0.950-1.737) | .10 |
| Current household composition | | .23 |
| Live alone | 1 (Ref) |  |
| Live with family | 1.258 (0.947-1.672) | .11 |
| Live with other people | 1.102 (0.732-1.658) | .64 |
| Chronic physical conditions |  |  |
| Obesity |  |  |
| - Yes | 0.723 (0.520-1.006) | .05 |
| - No | 1 (Ref) |  |
| Hypertension |  |  |
| - Yes | 0.918 (0.646-1.305) | .64 |
| - No | 1 (Ref) |  |
| Other chronic conditions |  |  |
| - Yes | **1.450 (1.109-1.895)** | **.007*** |
| - No | 1 (Ref) |  |
| Chronic mental illnesses |  |  |
| - Yes | **1.388 (1.087-1.773)** | **.009*** |
| - No | 1 (Ref) |  |

|  | Multiple logistic regression | |
| --- | --- | --- |
|  | aOR (95% C.I.) | *P* |
| Age (years) | **1.029 (1.017-1.041)** | **<.001*** |
| Gender |  |  |
| Male | Ref |  |
| Female | **1.256 (1.033-1.527)** | **.02*** |
| Race |  | **<.001*** |
| Asian | 1 (Ref) |  |
| White | **2.776 (1.892-4.073)** | **<.001*** |
| Black | **0.656 (0.458-0.939)** | **.02*** |
| American Indian or Alaska Native | 0.861 (0.542-1.368) | .53 |
| Others | 1.272 (0.960-1.685) | .09 |
| Working Status |  | .96 |
| Full-time employed | 1 (Ref) |  |
| Part-time/ self employed | 0.916 (0.657-1.277) | .60 |
| Student | 0.966 (0.754-1.239) | .79 |
| Others | 0.998 (0.647-1.540) | .99 |
| Years of education |  | .54 |
| 0-9 years | 1 (Ref) |  |
| 10-12 years | 0.817 (0.468-1.423) | .48 |
| More than 12 years | 0.763 (0.456-1.276) | .30 |
| Residence |  | **<.001*** |
| Urban area | 1 (Ref) |  |
| Rural area | **1.664 (1.327-2.087)** | **.001*** |
| Rural-urban fringe | 1.155 (0.842-1.584) | .37 |
| Current household composition | | **<.001*** |
| Live alone | 1 (Ref) |  |
| Live with family | **1.640 (1.227-2.193)** | **.001*** |
| Live with other people | 1.069 (0.709-1.611) | .75 |
| Chronic physical conditions |  |  |
| Obesity |  |  |
| - Yes | 0.726 (0.503-1.048) | .09 |
| - No | 1 (Ref) |  |
| Hypertension |  |  |
| - Yes | 1.251 (0.830-1.885) | .29 |
| - No | 1 (Ref) |  |
| Other chronic conditions |  |  |
| - Yes | **1.464 (1.091-1.964)** | **.011*** |
| - No | 1 (Ref) |  |
| Chronic mental illnesses |  |  |
| - Yes | 0.987 (0.764-1.274) | .92 |
| - No | 1 (Ref) |  |

**Supplementary table 4c** – Association between demographic factors and avoiding social events

|  | Multiple logistic regression | |
| --- | --- | --- |
|  | aOR (95% C.I.) | *P* |
| Age (years) | **0.989 (0.979-0.999)** | **.04*** |
| Gender |  |  |
| Male | Ref |  |
| Female | **1.443 (1.199-1.738)** | **<.001*** |
| Race |  | **.01*** |
| Asian | 1 (Ref) |  |
| White | 1.033 (0.753-1.418) | .84 |
| Black | **0.658 (0.459-0.944)** | **.02*** |
| American Indian or Alaska Native | 0.683 (0.428-1.090) | .11 |
| Others | **0.689 (0.526-0.904)** | **.007*** |
| Working Status |  | **.005*** |
| Full-time employed | 1 (Ref) |  |
| Part-time/ self employed | 0.741 (0.546-1.006) | .06 |
| Student | **1.318 (1.041-1.670)** | **.02*** |
| Others | 0.837 (0.571-1.226) | .36 |
| Years of education |  | .88 |
| 0-9 years | 1 (Ref) |  |
| 10-12 years | 1.022 (0.619-1.686) | .93 |
| More than 12 years | 1.079 (0.680-1.711) | .75 |
| Residence |  | .21 |
| Urban area | 1 (Ref) |  |
| Rural area | 1.034 (0.589-1.816) | .91 |
| Rural-urban fringe | 1.171 (0.636-2.159) | .61 |
| Current household composition | | .11 |
| Live alone | 1 (Ref) |  |
| Live with family | 0.871 (0.655-1.157) | .34 |
| Live with other people | 0.774 (0.513-1.165) | .22 |
| Chronic physical conditions |  |  |
| Obesity |  |  |
| - Yes | **0.693 (0.499-0.961)** | **.03*** |
| - No | 1 (Ref) |  |
| Hypertension |  |  |
| - Yes | 1.335 (0.937-1.903) | .11 |
| - No | 1 (Ref) |  |
| Other chronic conditions |  |  |
| - Yes | **1.438 (1.102-1.878)** | **.007*** |
| - No | 1 (Ref) |  |
| Chronic mental illnesses |  |  |
| - Yes | 1.125 (0.882-1.434) | .34 |
| - No | 1 (Ref) |  |

**Supplementary table 4d** – Association between demographic factors and feeling highly stressful

**Supplementary table 5a** Heat map of all factors


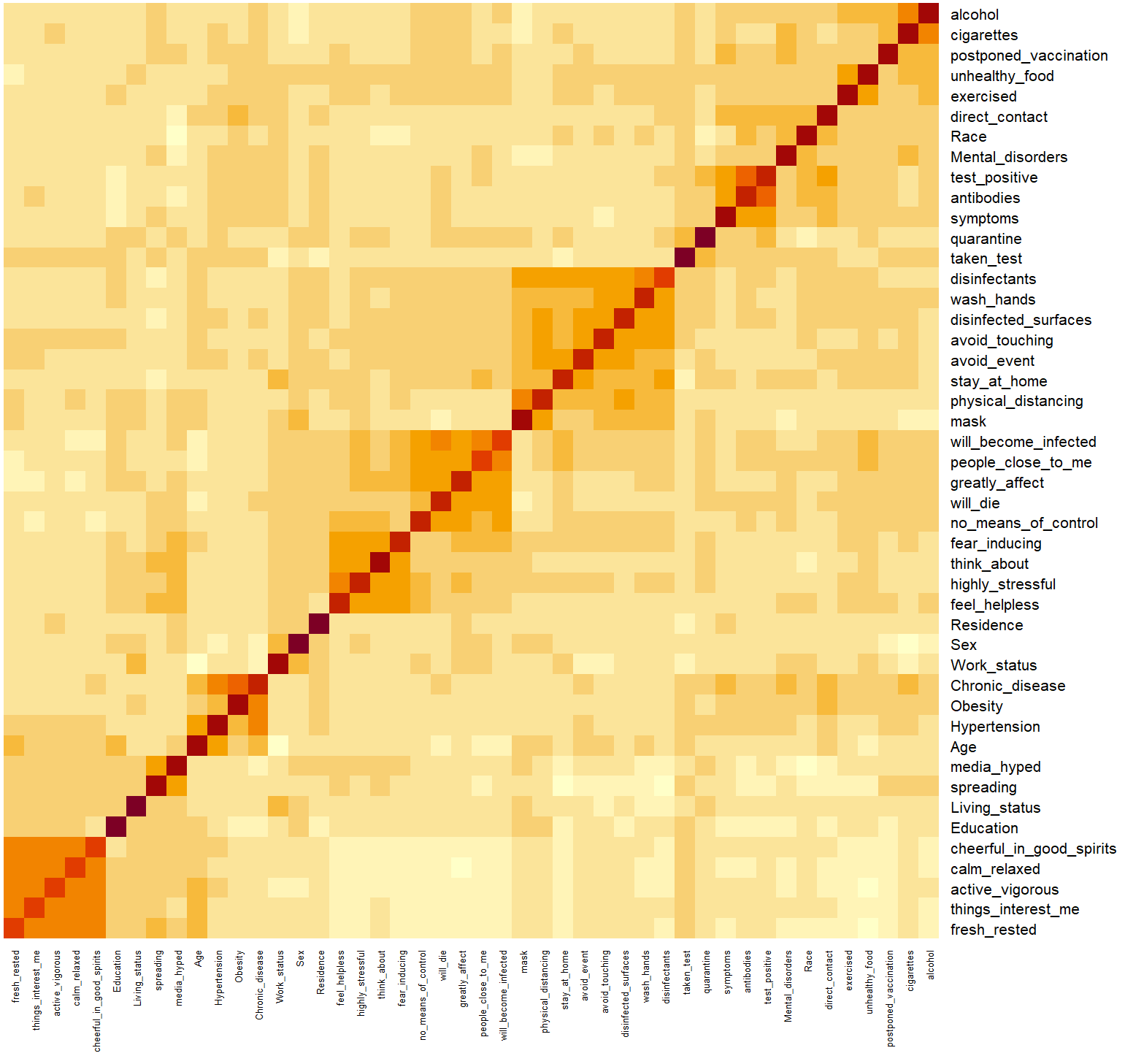


**Supplementary table 5b** Tree-like hierarchical clustering lines of all factors


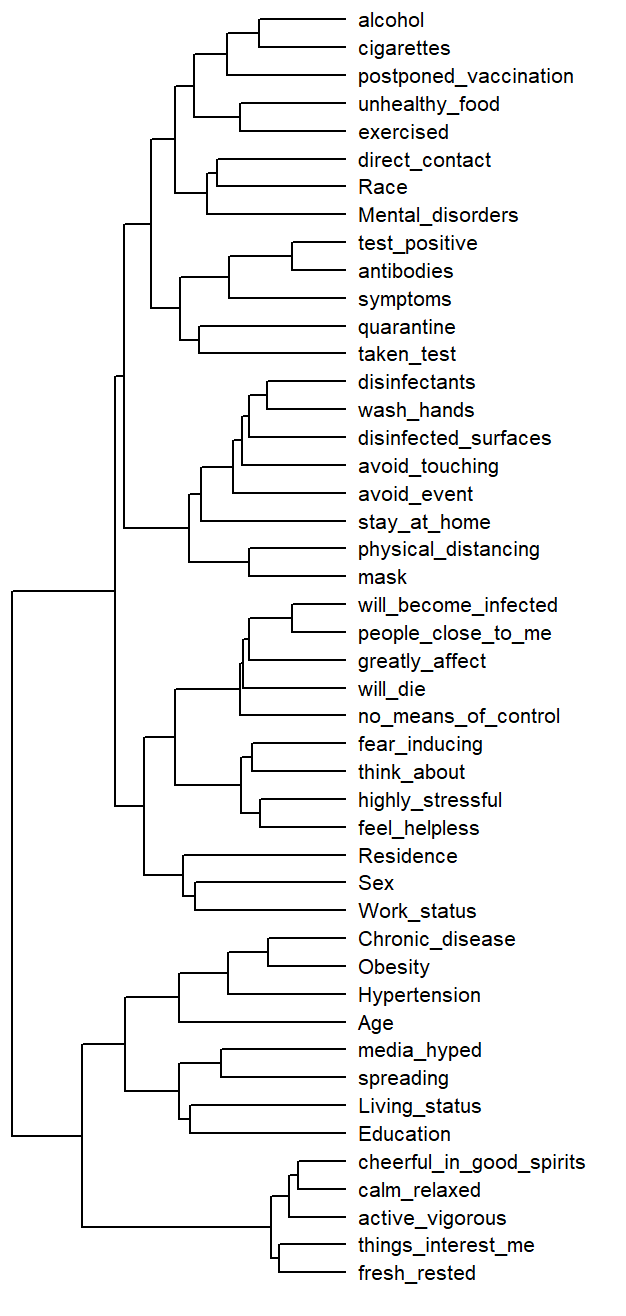


**Descriptions on heatmap and tree-like hierarchical clustering lines**

A heatmap is a graphical representation of data where values are depicted by color. The more intense the color in a cell, the higher the value. In our study, correlograms were plotted to show the associations among the variables. Each cell depicts the relationship between the intersecting variables by correlation coefficient.

A tree-like hierarchical clustering is a way of arranging variables in a hierarchy based on the distance or similarity between them. It consists of stacked branches (called clades) that break down into further smaller branches. At the lowest level will be individual variables and then they are grouped according to attributes into clusters with fewer and fewer clusters on higher levels. The end of each clade (called a leaf) is the data. The arrangement of the clades reveals how similar they are to each other. Two leaves in the same clade are more similar than two leaves in another clade. The length of the branch shows how close clusters are from one another. The longer the branch, the further and more different the clusters are.

**Supplementary table 6** Socio-demographic factors associated with increased weight in the past six months (sensitivity analysis with regions)

| Variables | | Multiple Logistic Regression analysis  aOR (95%CI) | *P** |  | |  |
| --- | --- | --- | --- | --- | --- | --- |
| **Regions** | |  |  |  | |  |
|  | Americas | 1 (ref) |  |  | |  |
|  | Asia Pacific | 0.78 (0.54 - 1.12) | .18 |  | |  |
|  | European | 0.25 (0.03 - 2.14) | .21 |  | |  |
|  | Middle East | 2.27 (0.75 - 6.89) | .15 |  | |  |
| **Age years** | | 0.97 (0.96 - 0.98) | <.001* |  | |  |
| **Sex** | | | | |  |  |
|  | Male (ref) | 1 (ref) |  |  | |  |
|  | Female | 1.16 (0.96 - 1.40) | .12 |  | |  |
| **Race** | |  |  |  | |  |
|  | Asian (ref) | 1 (ref) | .25 |  | |  |
|  | White | 1.06 (0.68 - 1.65) | .80 |  | |  |
|  | Black | 0.69 (0.44 - 1.08) | .10 |  | |  |
|  | American Indian or Alaska Native | 0.72 (0.42 - 1.24) | .24 |  | |  |
|  | Others | 0.89 (0.62 - 1.29) | .55 |  | |  |
| **Years of Education** | | | | |  |  |
|  | 0-9 years (ref) | 1 (ref) | .04* |  | |  |
|  | 10-12 years | 1.52 (0.89 - 2.58) | .12 |  | |  |
|  | > 12 years | 1.80 (1.10 - 2.96) | .02* |  | |  |
| **Residence** | | | | |  |  |
|  | Urban area (ref) | 1 (ref) | .28 |  | |  |
|  | Rural area | 1.11 (0.90 - 1.37) | .33 |  | |  |
|  | Rural–urban fringe | 1.24 (0.92 - 1.68) | .16 |  | |  |
| **Current household composition** | | | | |  |  |
|  | live alone (ref) | 1 (ref) | .07 |  | |  |
|  | live with family | 1.37 (1.02 - 1.84) | .04* |  | |  |
|  | live with other people | 1.12 (0.74 - 1.70) | .58 |  | |  |
| **Work/study status** | | | | |  |  |
|  | Full-time (ref) | 1 (ref) | .001* |  | |  |
|  | Part-time/self employed | 0.58 (0.42 - 0.80) | .001* |  | |  |
|  | Students | 0.70 (0.55 - 0.89) | .004* |  | |  |
|  | Others | 0.65 (0.44 - 0.98) | .04* |  | |  |
| **Chronic physical conditions** | | | | |  |  |
|  | Obesity |  |  |  | |  |
|  | Yes | 2.61 (1.88 - 3.63) | <.001 |  | |  |
|  | No | 1 (ref) |  |  | |  |
|  | Hypertension |  |  |  | |  |
|  | Yes | 1.17 (0.82 - 1.67) | .39 |  | |  |
|  | No | 1 (ref) |  |  | |  |
|  | Others |  |  |  | |  |
|  | Yes | 1.06 (0.81 - 1.37) | .69 |  | |  |
|  | No | 1 (ref) |  |  | |  |
| **Mental illnesses** | | | | |  |  |
|  | Yes | 0.82 (0.64 - 1.04) | .10 |  | |  |
|  | No (ref) | 1 (ref) |  |  | |  |
| *Significant at *p* < 0.05 | | | | |  |  |

**Supplementary table 7** Survey instruments

| Question | Options |
| --- | --- |
| * 1. Have you ever filled out this questionnaire? | - I have ever filled out it - I have never filled out it |
| * 2. Please note that you can stop the survey at any time. This will not entail any penalty, and it will not affect the services (health care services or others) that you receive. By selecting the “agree box”, you are agreeing that you are at least 18 years old, that you have read the information about the study, and that you voluntarily agree to take part in it. | - I agree to participate in this study - I do not agree to participate in this study |
| * 3. Date | Date/Time |
| **Part 1: Socio-demography** | |
| 4. How old are you? |  |
| 5. What is your sex? | - Male - Female - Other - I prefer not to tell |
| 6. Which country or region are you currently living in? | - Australia - Thailand - Canada - China - South Korea - Germany - Japan - India - Malaysia - Philippines - Mexico - New Zealand - Ecuador - United Kingdom - United States - Other (please specify) |
| 7. What is your race? | - American Indian or Alaska Native - Asian - Black or African American - Native Hawaiian or Other Pacific Islander - White - Other (please specify) |
| 8. How many years of education have you completed (including non-formal education such as part time, night)? | - 0-9 years - 10-12 years (secondary school completed) - more than 12 years |
| 9. Where do you live? | - Rural area - Urban area - Rural–urban fringe - Other (please specify) |
| 10. Who lives in your household besides yourself? | - I live alone - I live with my family members (parents, husband/wife, children, etc) - None of the above |
| 11. Please indicate your work or study status. | - Full-time employed - Part-time employed - Self-employed - Not employed but not student - Student - Retired - Caregiver - Others |
| 12. Please assess your weight change over the past six months: | - Increased - Remains the same - Decreased - Don’t know |
| 13. Please indicate which of the following conditions apply to you (Yes/no) | - Cardiovascular disease (e.g. coronary heart disease, heart failure, cardiomyopathy) - Hypertension - Type 2 Diabetes - Immunodeficiency, or taking medication that suppresses the immune system (e.g. corticosteroid) - Chronic disease of the respiratory system (e.g. asthma, chronic bronchitis) - Chronic liver disease - Chronic kidney disease - Cancer during past 5 years - Sickle cell disease - Obesity (Body mass index ≥30 kg/m2) |
| 14. Have you ever been diagnosed by a doctor or therapist with one or more of the following? (Yes/ No) | - Depression - Mania/Bipolar disorder - Psychotic disorders (including schizophrenia) - Anxiety disorder - Posttraumatic stress disorder - Eating disorder - Compulsive disorders (OCD) - Substance abuse or Addiction disorder - Attention disorder (ADD or ADHD) - Somatoform disorder - Personality disorder - Autism Spectrum - Disorder (including Asperger’s Syndrome) - Cognitive disorder/dementia |
| **Part 2: COVID-19 personal experience** | |
| 15. Do you currently suffer from COVID-19 symptoms such as fever, dry cough, breathing problems, sore throat, loss of smell/taste, headaches or diarrhoea? | - Yes - No |
| 16. Have you taken any test for COVID-19? | - Yes - No |
| 17. Have you been tested positive for COVID-19? | - Yes - No |
| 18. Have you been tested positive for COVID-19 antibodies? | - Yes - No |
| 19. Has anyone with whom you have had direct contact in the past two weeks become infected with COVID-19 that you are aware of? | - Yes - No |
| 20. Please indicate whether you have been in quarantine due to Covid-19. | - Yes - No |
| **Part 3: Health behaviors** | |
| 21. Over the past 14 days I...  (Strongly disagree, Disagree, Neutral, Agree, Strongly agree, N/A) | …Have consumed substantially more alcohol than usual.  …Have smoked considerably more cigarettes than usual.  …Exercised less than I did before the pandemic.  …Ate more unhealthy food than I did before the pandemic (such as fried food, coke, etc).  …Postponed vaccination for myself or my child. |
| 22. During the last 14 days, which of the following measures have you taken to prevent infection from COVID-19? (Strongly disagree, Disagree, Neutral, Agree, Strongly agree, N/A) | - Frequently washed my hands with soap and water for at least 20 seconds.  - Avoided touching my eyes, nose and mouth with unwashed hands.  - Used disinfectants/sanitizer to clean hands when soap and water were not available.  - Avoided a social event I wanted to attend.  - Stayed at home from work/school.  - Wore a mask in public.  - Ensured physical distancing in public.  - Disinfected surfaces. |
| **Part 4: Mental Health** | |
| 23. Please choose one option per row below. COVID-19 to me feels ...  (Strongly disagree, Disagree, Neutral, Agree, Strongly agree) | …Spreading slowly  …Something I think about all the time  …Fear-inducing  …Media hyped  …Something that makes me feel helpless  …Highly stressful |
| 24. How do you currently perceive the risk of the COVID-19 pandemic? “I am worried that…”  (Strongly disagree, Disagree, Neutral, Agree, Strongly agree) | …have no means of control over the COVID-19 pandemic.  …I will become infected with COVID-19.  …People close to me are going to be infected with COVID-19.  …The consequences of the COVID-19 pandemic will greatly affect me personally.  …I will die of COVID-19. |
| 25. We would now like for you to indicate your general well-being: Over the past 14 days…  (All of the time, Most of the time, More than half the time, Less than half the time, Some of the time, At no time) | … I have felt cheerful and in good spirits.  … I have felt calm and relaxed.  … I have felt active and vigorous.  … I have woken up feeling fresh and rested.  … my daily life has been filled with things that interest me. |

**Supplementary material 1**

* 1. Have you ever filled out this questionnaire?

I have ever filled out it

I have never filled out it

* 2. Please note that you can stop the survey at any time. This will not entail any penalty, and it will not affect the services (health care services or others) that you receive. By selecting the “agree box”, you are agreeing that you are at least 18 years old, that you have read the information about the study, and that you voluntarily agree to take part in it.

I agree to participate in this study

I do not agree to participate in this study

* 3. Date

Date/Time

**Part 1: Socio-demography**

4. How old are you?

5. What is your sex?

Male

Female

Other

I prefer not to tell

6. Which country or region are you currently living in?

Australia

Thailand

Canada

China

South Korea

Germany

Japan

India

Malaysia

Philippines

Mexico

New Zealand

Ecuador

United Kingdom

United States

Other (please specify)

7. What is your race?

American Indian or Alaska Native

Asian

Black or African American

Native Hawaiian or Other Pacific Islander

White

Other (please specify)

8. How many years of education have you completed (including non-formal education such as part time,

night)?

0-9 years

10-12 years (secondary school completed)

more than 12 years

9. Where do you live?

Rural area

Urban area

Rural–urban fringe

Other (please specify)

10. Who lives in your household besides yourself?

I live alone

I live with my family members (parents, husband/wife, children, etc)

None of the above

11. Please indicate your work or study status.

Full-time employed

Part-time employed

Self-employed

Not employed but not student

Student

Retired

Caregiver

Others

12. Please assess your weight change over the past six months:

Increased

Remains the same

Decreased

Don’t know

13. Please indicate which of the following conditions apply to you (Yes/no)

Cardiovascular disease (e.g. coronary heart disease, heart failure, cardiomyopathy)

Hypertension

Type 2 Diabetes

Immunodeficiency, or taking medication that suppresses the immune system (e.g. corticosteroid)

Chronic disease of the respiratory system (e.g. asthma, chronic bronchitis)

Chronic liver disease

Chronic kidney disease

Cancer during past 5 years

Sickle cell disease

Obesity (Body mass index ≥30 kg/m2)

14. Have you ever been diagnosed by a doctor or therapist with one or more of the following? (Yes/ No)

Depression

Mania/Bipolar disorder

Psychotic disorders (including schizophrenia)

Anxiety disorder

Posttraumatic stress disorder

Eating disorder

Compulsive disorders (OCD)

Substance abuse or Addiction disorder

Attention disorder (ADD or ADHD)

Somatoform disorder

Personality disorder

Autism Spectrum

Disorder (including Asperger’s Syndrome)

Cognitive disorder/dementia

**Part 2: COVID-19 personal experience**

15. Do you currently suffer from COVID-19 symptoms such as fever, dry cough, breathing problems, sore throat, loss of smell/taste, headaches or diarrhoea?

Yes

No

16. Have you taken any test for COVID-19?

Yes

No

17. Have you been tested positive for COVID-19?

Yes

No

18. Have you been tested positive for COVID-19 antibodies?

Yes

No

19. Has anyone with whom you have had direct contact in the past two weeks become infected with COVID-19 that you are aware of?

Yes

No

20. Please indicate whether you have been in quarantine due to Covid-19.

Yes

No

**Part 3: Health behaviors**

21. Over the past 14 days I... (Strongly disagree, Disagree, Neutral, Agree, Strongly agree, N/A)

Have consumed substantially more alcohol than usual.

Have smoked considerably more cigarettes than usual.

Exercised less than I did before the pandemic.

Ate more unhealthy food than I did before the pandemic (such as fried food, coke, etc).

Postponed vaccination for myself or my child.

22. During the last 14 days, which of the following measures have you taken to prevent infection from COVID-19? (Strongly disagree, Disagree, Neutral, Agree, Strongly agree, N/A)

Frequently washed my hands with soap and water for at least 20 seconds.

Avoided touching my eyes, nose and mouth with unwashed hands.

Used disinfectants/sanitizer to clean hands when soap and water were not available.

Avoided a social event I wanted to attend.

Stayed at home from work/school.

Wore a mask in public.

Ensured physical distancing in public.

Disinfected surfaces.

**Part 4: Mental Health**

23. Please choose one option per row below. COVID-19 to me feels ... (Strongly disagree, Disagree, Neutral, Agree, Strongly agree)

Spreading slowly

Something I think about all the time

Fear-inducing

Media hyped

Something that makes me feel helpless

Highly stressful

24. How do you currently perceive the risk of the COVID-19 pandemic? “I am worried that…” (Strongly disagree, Disagree, Neutral, Agree, Strongly agree)

have no means of control over the COVID-19 pandemic.

I will become infected with COVID-19.

People close to me are going to be infected with COVID-19.

The consequences of the COVID-19 pandemic will greatly affect me personally.

I will die of COVID-19.

25. We would now like for you to indicate your general well-being: Over the past 14 days… (All of the time, Most of the time, More than half the time, Less than half the time, Some of the time, At no time)

… I have felt cheerful and in good spirits.

… I have felt calm and relaxed.

… I have felt active and vigorous.

… I have woken up feeling fresh and rested.

… my daily life has been filled with things that interest me.
